# The effect of reduced gravity on cryogenic nitrogen boiling and pipe chilldown

**DOI:** 10.1038/npjmgrav.2016.33

**Published:** 2016-10-13

**Authors:** Samuel Darr, Jun Dong, Neil Glikin, Jason Hartwig, Alok Majumdar, Andre Leclair, Jacob Chung

**Affiliations:** 1 Department of Mechanical and Aerospace Engineer, University of Florida, Gainesville, FL, USA; 2 Power and In-Space Propulsion Branch, NASA Glenn Research Center, Cleveland, OH, USA; 3 NASA Marshall Spaceflight Center, Huntsville, AL, USA

## Abstract

Manned deep space exploration will require cryogenic in-space propulsion. Yet, accurate prediction of cryogenic pipe flow boiling heat transfer is lacking, due to the absence of a cohesive reduced gravity data set covering the expected flow and thermodynamic parameter ranges needed to validate cryogenic two-phase heat transfer models. This work provides a wide range of cryogenic chilldown data aboard an aircraft flying parabolic trajectories to simulate reduced gravity. Liquid nitrogen is used to quench a 1.27 cm diameter tube from room temperature. The pressure, temperature, flow rate, and inlet conditions are reported from 10 tests covering liquid Reynolds number from 2,000 to 80,000 and pressures from 80 to 810 kPa. Corresponding terrestrial gravity tests were performed in upward, downward, and horizontal flow configurations to identify gravity and flow direction effects on chilldown. Film boiling heat transfer was lessened by up to 25% in reduced gravity, resulting in longer time and more liquid to quench the pipe to liquid temperatures. Heat transfer was enhanced by increasing the flow rate, and differences between reduced and terrestrial gravity diminished at high flow rates. The new data set will enable the development of accurate and robust heat transfer models of cryogenic pipe chilldown in reduced gravity.

## Introduction

Future orbiting propellant depots and human-carrying orbital transfer vehicles to Mars will need to utilize the high thrust and efficiency of liquid cryogenic chemical propulsion or nuclear thermal propulsion.^1–4^ The transfer of cryogenic propellants in space, however, has yet to be accomplished, partly owing to the limited availability of cryogenic two-phase heat transfer data in reduced gravity.^4^ When a cryogenic propellant like liquid hydrogen or liquid oxygen is transferred from a supply tank to an engine or receiver tank, a transient two-phase heat transfer process ensues where the cryogen boils into vapor until the transfer pipe and the receiving hardware are cooled to the liquid temperature. After this “chilldown” process, single-phase liquid can be transferred to the destination. The consumed propellant during chilldown generates substantial vapor that must be vented overboard. The ability to accurately simulate chilldown is necessary for designing efficient in-space cryogenic propulsion systems that minimize this propellant consumption. But it has been shown that the longstanding correlations to predict heat transfer between the two-phase boiling fluid and the pipe wall that were developed from data with water and refrigerants do not work with cryogens.^5^ Thus, there is a current lack of accurate heat transfer correlations for cryogenic chilldown.

Experimental studies of cryogenic chilldown of a transfer pipe in terrestrial gravity (1-g) have been carried out since the 1960s.^6,7^ But only recently has there been a thorough experimental investigation of the entire chilldown process in 1-g that reports the measured heat transfer coefficients for all boiling regimes over a large range of conditions.^8,9^ A similar experimental study covering a large range of conditions is needed for reduced gravity. Two-phase boiling data with water, refrigerants, and other non-cryogenic liquids in reduced gravity have shown that the lack of gravity significantly alters the flow patterns and heat transfer from what is seen in 1-g.^10,11^ The general observations from these experiments show that the absence of a buoyancy force in reduced gravity lowers the heat transfer because the only mechanism available to sweep vapor bubbles away from the surface is the drag and inertial forces from the bulk fluid motion. Vapor bubbles generated at the surface can more easily coalesce to form an insulating barrier between the liquid phase and the wall. The sparse available cryogenic chilldown data in reduced gravity also reveal similar trends.^12–14^

Because of the large difference in density between the vapor and liquid phases, buoyancy forces have a major role in flow boiling under 1-g. Different flow patterns, which determine the wall-to-fluid heat transfer, are expected depending on the flow orientation with respect to the gravity vector. Horizontal flows (perpendicular to gravity) are stratified, with the denser liquid near the bottom and lighter vapor near the top.^15,16^ Vertical flows exhibit axisymmetric flow patterns. During film boiling at high mass fluxes, inverted annular flow occurs, with a liquid core surrounded by a vapor annulus, creating an insulating gap between the liquid and pipe wall.^17–19^ In upward flow, buoyancy forces assist vapor motion relative to the bulk fluid motion, increasing convection heat transfer, whereas for downward flow the same buoyancy force slows the vapor relative to the bulk fluid motion, reducing the convection heat transfer.^9^ As mass flux increases, this effect diminishes as forced convection from bulk fluid motion dominates over natural convection, and the flow patterns and heat transfer are nearly the same.

The flow patterns in reduced gravity conditions can also be distinguished from those seen in 1-g. Like vertical flow orientations, the flow patterns are generally axisymmetric, but as surface tension becomes more relevant in reduced gravity, bubbles coalesce more easily. The lack of buoyancy forces lessens the interfacial velocity so that interfacial waves are less intense and droplet and bubble breakup are less frequent. The result is flow patterns that have much larger vapor patches or liquid slugs. In 1-g conditions, the flow patterns are very tortuous and the liquid and vapor are broken into small droplets and bubbles, whereas microgravity flow boiling experiences large continuous patches of the two phases with smooth interfaces.^10,20^

The existing two-phase flow boiling data in reduced gravity consist primarily of nucleate boiling heat transfer and critical heat flux measurements.^10,11^ Only a few quenching studies in reduced gravity conditions exist that cover the entire chilldown process, including film boiling heat transfer and the rewetting temperature.

Adham-Khodaparast *et al*.^21^ investigated film boiling and rewetting for the quenching of a hot flat surface with flowing R113. Lower heat transfer rates were reported during reduced gravity as compared with normal gravity, which contributed to the thickening of the vapor layer. The wall superheat and heat flux at the onset of rewetting and the critical heat flux were found to increase with the inlet liquid subcooling, mass flux, and gravity level. The effect of gravity was more important for low flow rates and less relevant for high flow rates.

Westbye *et al.* reported quenching of a hot thin-walled stainless steel tube by injection of subcooled R113 into the tube under both 1-g (horizontal flow direction) and reduced gravity.^22^ The rewetting temperatures were 15 to 25 K lower in reduced gravity than those obtained in 1-g, and the film boiling heat transfer coefficients in reduced gravity were only 20–50% of those in 1-g tests. It was also reported that the rewetting velocity was slightly greater in reduced gravity. Nucleate and transition boiling under reduced gravity shifted to lower wall superheats as compared with 1-g. Nucleate flow boiling conditions were similar regardless of gravity level. Compared with 1-g, maximum heat flux in reduced gravity was lower at low flow rates and higher at high flow rates; the crossover occurred at a liquid Reynolds number (Re) of 8,300. Finally, the heat transfer coefficient was consistently higher in 1-g conditions.

Celata *et al*.^20,23^ reported experimental results for 1-g (upward flow) and reduced gravity (10^−2^ g) chilldown using FC-72 during flying parabolic trajectories. The results showed a significant decrease in the quenching front velocity in reduced gravity, whereas the rewetting temperature was not affected by the gravity levels. The flow patterns were inverted annular and bubbly flows in reduced gravity.

For cryogenic flow boiling in reduced gravity, there is very little heat transfer data reported owing to experimental difficulties. Antar,^24^ Antar and Collins,^12,25,26^ Antar *et al.*,^27^ and Sathasivam^28^ all reported qualitative results and analysis from the same cryogenic chilldown study onboard a KC-135 aircraft in reduced gravity using liquid nitrogen (LN_2_). Comparison of the pipe wall temperature measurement versus time were made between one test from the flight experiment and a 1-g experiment in the upward flow direction. During film boiling, the flow pattern was a sputtering leading core followed by a liquid filament annular flow regime. This flow regime was composed of a long liquid column flowing in the center of the tube surrounded by a thick vapor layer. The filamentary flow was attributed to the lack of difference in the speed of vapor and liquid phases. The tube wall cooling rate was diminished and the quenching front speed was slower in low gravity.

Yuan *et al*.^13^ conducted experiments and extensive analysis to characterize the behavior of laminar LN_2_ flows within a horizontal tube under 1-g and reduced gravity in a 1.8 s drop tower. Heat transfer was smaller in reduced gravity owing to inverted annular flow. The bottom wall temperature dropped more quickly than the upper wall temperature in 1-g. The buoyancy force enforced quicker and more consistent liquid contact along the bottom wall.

Kawanami *et al*.^14^ studied the effects of heat transfer in a 0.7 cm ID vertical tube for LN_2_ under 1-g vertical flow and reduced gravity for liquid Re numbers between 4,500 and 14,000. The reduced gravity experiment was performed in a 10 s drop tower. The heat transfer increased by up to 20% in reduced gravity compared with 1-g, which was associated to a higher quench front velocity. The differences between 1-g and reduced gravity results decreased with increasing fluid velocity, suggesting a liquid Re number at which there is no difference in heat transfer characteristics. Interestingly, the results are in disagreement with the majority of other experiments, including current results. This disagreement in the historical studies and the minimal amount of data that have been presented make it difficult to provide generalizations about cryogenic chilldown in reduced gravity. Therefore, to further the understanding of cryogenic chilldown heat transfer and to make it possible to develop accurate heat transfer correlations, it is necessary that cryogenic chilldown data and analysis over a wide range of conditions be provided by a systematic experimental investigation, which is the purpose of this work.

The objective of the current work is to provide a wide ranging data set of reduced gravity cryogenic pipe chilldown data from an experiment using LN_2_ onboard a C9 aircraft. Heat transfer data were obtained from flowing LN_2_ through a short, stainless steel pipe equipped with all the data acquisition needed for developing heat transfer correlations. This experiment was an extension of 1-g experiments reported in Darr *et al.*, which obtained data to support the development of heat transfer correlations for 1-g.^8,9^ Therefore, the current work minimizes buoyancy forces from the chilldown process so that these correlations can be extended to reduced gravity conditions. The correlations developed from these data can be integrated into fluid network simulation software and used as a tool for designing in-space cryogenic propulsion systems.

## Results

A total of 10 successful chilldown tests were completed in the reduced gravity experiment. The fastest chilldown time to achieve saturated temperature at the downstream TC station was about 13 s. A majority of the tests, however, did not completely chill down in the 20–23 s window of reduced gravity. Nonetheless, accurate film boiling data were obtained during all reduced-gravity periods. Along with this article is included a Supplementary Excel File that contains the mass flux, wall temperature, and pressure histories for each test.

The 1-g chilldown experiments were carried out with the new rig at similar conditions to most of the reduced gravity tests to provide direct comparisons and facilitate the understanding of the effects of gravity on chilldown. Tests were completed in the upward, downward, and horizontal flow configurations. The entire rig, except for the dewar that was placed next to the rig, was flipped on either side from its normal resting position for the upward and downward flow tests. Several test attempts were necessary to closely match the conditions of each reduced gravity test because the backpressure was normally around 21 kPa due to the high altitude testing, whereas the backpressure for the 1-g tests was near a sea level pressure of 101 kPa.

Table 1 lists the time-averaged (over the duration of reduced gravity period) inlet liquid Re number (computed from Re_in_=*GD*/*μ*_l_, where *G* is the time-averaged mass flux, which is the mass flow rate divided by the inner cross-sectional area of the test section, *D* is the inner diameter of the test section, and *μ*_l_ is the saturated liquid dynamic viscosity based on the time-averaged inlet pressure), time-averaged pressure at the upstream TC station, time-averaged degree of inlet subcooling, and time-averaged mass flux for both the reduced gravity and 1-g tests. The subcooling is defined as the difference between the saturation temperature corresponding to the inlet pressure and the measured inlet temperature. If the value is positive, it means the liquid is subcooled. Subcooling was not achievable for any of the microgravity tests owing to the long time period of pressurizing the dewar, which would cause the liquid to boil in the line upstream of the test section before each test.

### Quality of reduced gravity experimental runs

**Figure 1:**
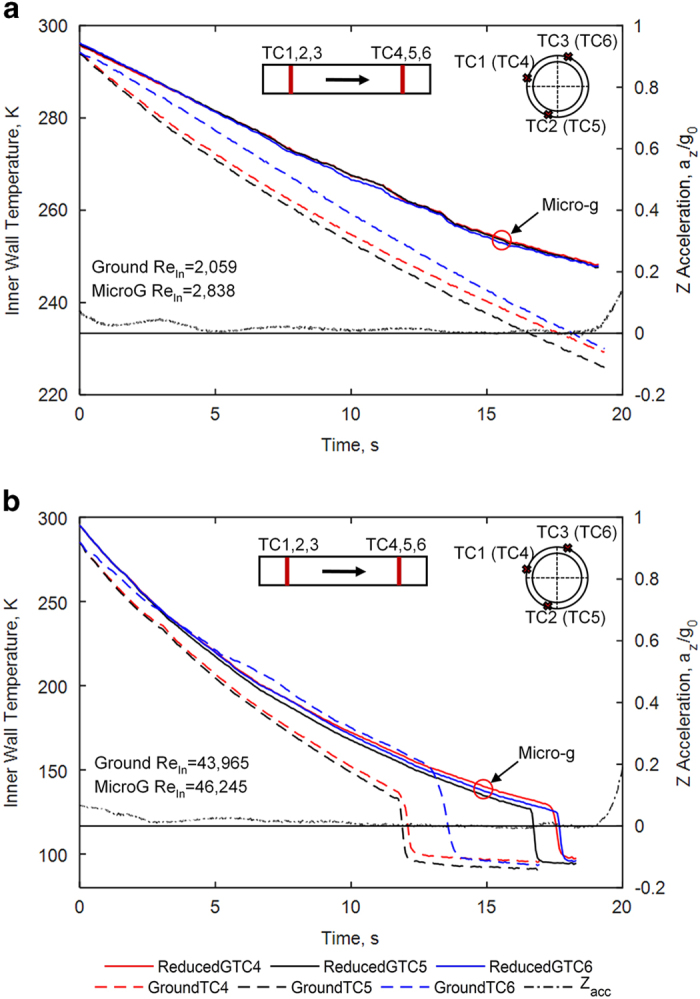
Quality of reduced gravity. Comparison of chilldown curves at the downstream TC station (TC4–6) between reduced gravity and horizontal flow in 1-g at (**a**) low Re_In_ (case 1) and (**b**) high Re_In_ (case 9). The presence of gravity causes the bottom and side thermocouples to chill down faster than the top. In reduced gravity, the chilldown rate is the same over the entire cross-section because of the axisymmetric flow patterns. Low-flow cases did not achieve full chilldown to liquid temperature before the end of the reduced-gravity period. The acceleration in the *z* direction (upward in the aircraft’s point of view) normalized by *g*_0_=9.81 m/s^2^ is given on the secondary *y* axis to show the quality of reduced gravity.

The quality of the reduced-gravity environment provided by the C9 parabolic flights is demonstrated in Figure 1, which represent cases at low Re_in_ (Figure 1a—case 1) and high (Figure 1b—case 9) Re_in_, respectively. Two sets of chilldown curves at the downstream TC station are plotted for similar Re_in_ in each figure. One set was collected in reduced gravity and the other was in 1-g in the horizontal flow configuration. Each set is composed of three curves that represent the local temperature histories registered by the thermocouples located on the top (TC6), side (TC4), and bottom (TC5) tube surfaces at the same downstream TC station during chilldown. The z-acceleration (upward in the aircraft coordinate system) is also plotted versus time in the same figure, and it was very similar for each parabola. The first 5–10 s are within ±0.1 g, and the remainder of the reduced-gravity period is within ±0.02 g.

The pipe test section was mounted parallel to the plane floor so that the acceleration vector was always perpendicular to the flow direction. Any significant acceleration would cause the temperature at the bottom of the cross-section to chill down faster than the temperature at the top of the cross-section due to buoyancy, as seen in the horizontal 1-g tests. However, three temperature readings for the reduced gravity test are bundled closely together, which shows that the buoyancy force was negligible, resembling 0-g conditions. The temperature readings for the 1-g test are separated from one another, with the top wall showing the slowest chilldown rate and the bottom wall the fastest, which demonstrates the typical stratification effects due to gravity. The wavy feature of the chilldown curve at low Re_in_ is owing to pressure fluctuations caused by the two-phase boiling, shown similarly in 1-g experiments by Darr *et al.* ^8^ For the high-flow case, fluctuations are dominated by the high driving pressure, resulting in a smoother chilldown curve.

### Complete results for five cases at different Re_in_

**Figure 2:**
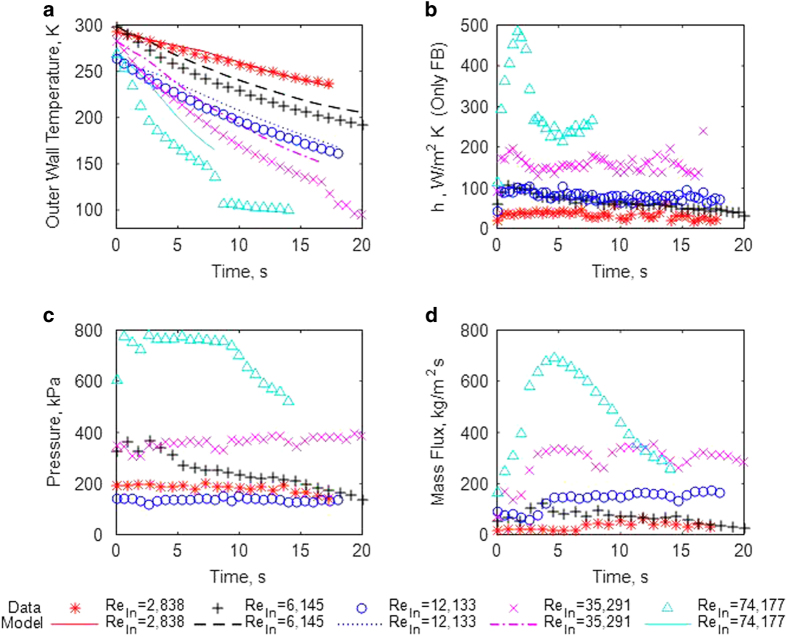
Reduced-gravity test comparison. Test section data and film boiling model prediction of reduced gravity tests at downstream TC station for several inlet Re_In_ (cases 1, 2, 4, 8, and 10). (**a**) Chilldown curve data and film boiling model prediction; (**b**) HTC data in only the film boiling regime; (**c**) pressure data; (**d**) mass flux data. In general, the higher the mass flux, the higher is the HTC owing to the increased convection.

Figures 2a–d plot results for chilldown curve, pressure history, mass flux history, and film boiling heat transfer coefficient (HTC) at the downstream TC station, respectively, for reduced-gravity cases 1, 2, 4, 8, and 10. (The computed HTC values for every test is included in the Supplementary Excel File.) As both time and gaseous nitrogen were limited during each flight, the test section was not always recovered to the target initial temperature of 293 K before each test run. For cases 8 and 10, the test section reached the rewetting temperature before the reduced-gravity period ended. The HTC data after the rewetting temperature were left out of Figure 2b because the large heat flux values during transition and nucleate boiling would make the scale of the figure too large to differentiate between the film boiling HTC values for all the cases. The HTC for two-phase boiling is defined as $h=q_{i}^{\prime \prime }/\left(T_{i}-T_{sat}\right)$, where *q*_i_″, *T*_i_, *T*_sat_, are the inner wall-to-fluid heat flux, inner wall temperature, and the saturation temperature of the fluid based on the local pressure, respectively. The inverse heat transfer solution given for an infinitely long cylinder was used to calculate *q*_i_″ and *T*_i_ from the measured outer wall temperature readings.^29^ Parasitic heat transfer from radiation and gas conduction are accounted for in the calculation of *q*_i_″. The overall uncertainty in *h* and *q*_i_″ in the film boiling heat transfer regime varies between 11 and 12.5% for all the tests. Details of the inverse heat transfer solution, parasitic heat transfer models, and uncertainty analysis on all the variables are in Darr *et al.*^8^

Figures 2a and b show faster chilldown rates and higher HTC at higher Re_in_, echoing trends in 1-g. Figure 2c displays the pressure at the downstream TC station for each test. The fluctuations with time are due to large density variations from phase change producing varying flow resistance. Figure 2d provides the mass flux history, which generally is higher for larger driving pressures. In general, an increase in the mass flux will enhance the heat transfer between fluid and pipe wall. However, for Case 10 the peaks of mass flux and film boiling HTC do not happen at the same time, but are separated by about 3.0 s. This may be owing to a small lag of the flow rate measurement and the actual flow rate at the test section as the flow meters are approximately 2 m downstream of the test section.

Also included for each chilldown test in Figure 2a is the wall temperature prediction of a numerical simulation that was developed earlier for 1-g downward flow.^30^ The numerical simulation was a one-dimensional finite volume solver of the energy equation of the test section pipe. The pipe was divided into axial nodes that each received energy source terms from the convective heat transfer of the LN_2_ flow on the inside of the tube and the parasitic heat transfer on the outside. Heat transfer correlations for the film boiling, transition boiling, and nucleate boiling regimes were developed to calculate the convective heat transfer at each node given the local conditions. Details of the numerical procedure and the correlations can be found in Darr *et al.*^30^ The results of the numerical simulation are only reported for the film boiling regime. The film boiling correlation used in the 1-g simulation was the sum of a two-phase turbulent convection term and a droplet-to-wall heat transfer term. From subsequent analysis of the 1-g upward data, 1-g downward data, and the recent reduced-gravity data, it was concluded that a better correlation could be achieved by including a third term that is constructed from the buoyancy-driven heat transfer correlation developed from Bromley.^31^ The correlation is

(1)hfb=htc+hdw+hb

(2)htc=c1[1+(DL+0.1D)0.7](kvD)(1-x)c2Revc3Prv0.4

(3)hdw=c4(klD)exp[-(LD)c5]Welc6Jal-1

(4)hb=c7[ρv(ρl-ρv)gHlv'kv34Lµv(Ti-Tsat)]1/4

where *L* is the axial length from the quenching front, *x* is the equilibrium quality, *g* is acceleration due to gravity, *k* is thermal conductivity, *ρ* is density, and subscripts v and l indicate vapor and liquid properties calculated at *T*_sat_, respectively. The vapor Reynolds and Prandtl numbers are Re_v_=*GD*/*μ*_v_ and Pr_v_=*C*_pv_ *μ*_v_/*k*_v_, respectively, and *C*_pv_ is the vapor specific heat at constant pressure. The liquid Weber number is We_l_=*G*^2^ *D*/(*ρ*_l_ *σ*_l_) and *σ*_l_ is the liquid surface tension. The liquid Jakob number is *Ja*_l_=*C*_pl_(*T*_i_–*T*_sat_)/*H*_lv_. The modified heat of vaporization, $H_{lv}^{'}=H_{lv}+0.375C_{pv}\left(T_{i}-T_{sat}\right)$, is used since all of the heat from the wall is not spent vaporizing the liquid, but instead some is spent superheating the vapor. The buoyancy term, equation (4), is significant at low Re_in_ for 1-g and is set to zero for reduced gravity. The turbulent convective term, equation (2), dominates at moderate Re_in_. The droplet-to-wall term, equation (3), becomes significant at high Re_in_ and begins to dominate at extremely high Re_in_. The coefficients c_1_ through c_7_ were determined by curve fitting the correlation to the data. They are given for each flow direction in Table 2.

Overall, the numerical simulation matches the chilldown curve in the film boiling regime with good accuracy for all the tests. Except for the lowest Re_in_ case, the chilldown rate is slightly under-predicted for the initial 2–3 s. After this period, the chilldown rate of the simulation matches that of the data. It is believed the reasoning behind this is that the initial readings of the mass flow rate meter are less than the actual mass flow rate in the test section. The burst of fluid entering the test section at the test start creates a large amount of flow in the test section that is not communicated downstream at the mass flow meter. After 2–3 s, a sufficiently steady mass flow is achieved through the entire system.

### Effects of gravity on chilldown heat transfer

**Figure 3:**
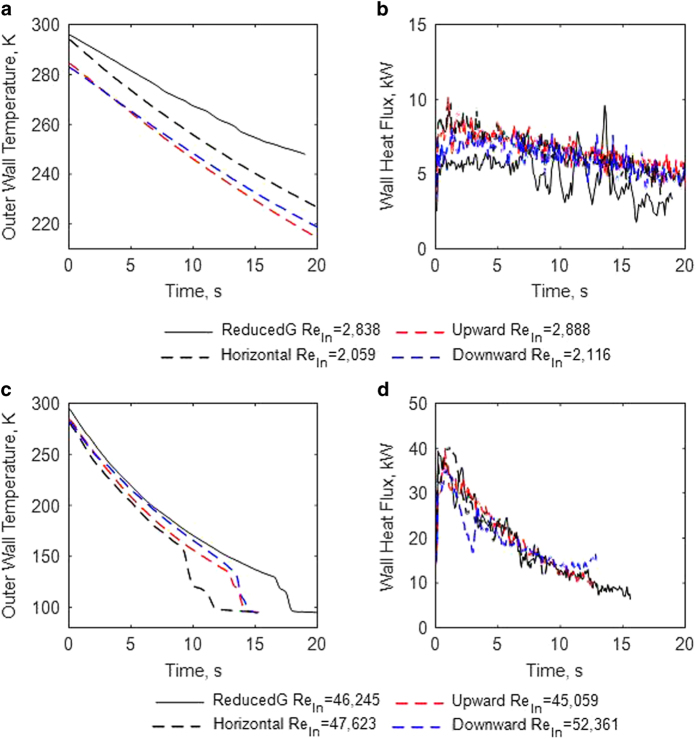
Effect of gravity. Comparison of chilldown curves between reduced gravity and 1-g at the downstream TC station at low Re_In_ (case 1) and high Re_In_ (case 9). (**a** and **c**), chilldown curve; (**b** and **d**) heat flux curve.

Figure 3 plots the upward, horizontal, and downward 1-g and reduced gravity chilldown curves at the downstream TC station. Figures 3b and d show the heat flux corresponding to Figures 3a and c, respectively. The reduced-gravity heat flux in Figure 3b is clearly smaller than each 1-g case. The average heat flux over this time period for the reduced-gravity test is 20–25% smaller than that for 1-g. However, the heat flux under the reduced gravity condition in Figure 3d is about the same as 1-g in all the flow directions. Comparing this difference between the low and high Re_in_ cases, the chilldown rate is more affected by the gravity level at lower flow rates, and the chilldown rate is significantly slower in reduced gravity than in 1-g for low flow rates. As the flow rate increases the chilldown rate difference decreases between reduced gravity and 1-g, consistent with historical trends. In Figure 3c, the rewetting temperature under reduced gravity is approximately 26 K lower than 1-g horizontal configuration, 5 K lower than 1-g upward configuration, and 3 K lower than 1-g downward configuration.

## Discussion

Results showed that the reduced gravity conditions during each test were high quality as there was negligible angular asymmetry in the pipe wall temperature compared with 1-g horizontal flow during chilldown. The increasing mass flow rate enhanced the wall-to-fluid heat transfer, which resulted in a faster chilldown rate. The film boiling heat transfer was notably smaller in reduced gravity than in 1-g at low flow rates. At Re_in_≈2,500 it was about 20 to 25% lower than that seen in 1-g. This implied that chilldown at low flow rates is significantly less efficient in reduced gravity compared to any 1-g flow direction. The physical cause of this is that the rapid, irregular interfacial oscillations that are normally present in 1 g and act to increase the heat transfer, are not present in 0 g. The effect of gravity diminished as the mass flow rate was increased. This can be explained by the forced convection heat transfer dominating over buoyancy effects at high mass flux. In addition, the rewet temperature occurred at slightly lower temperatures in reduced gravity when compared with 1-g upward and downward flows. Without gravity acting to increase interfacial oscillations, the liquid core has slightly less potential to break up into droplets and carry radial momentum towards the wall.

## Materials and Methods

**Figure 4:**
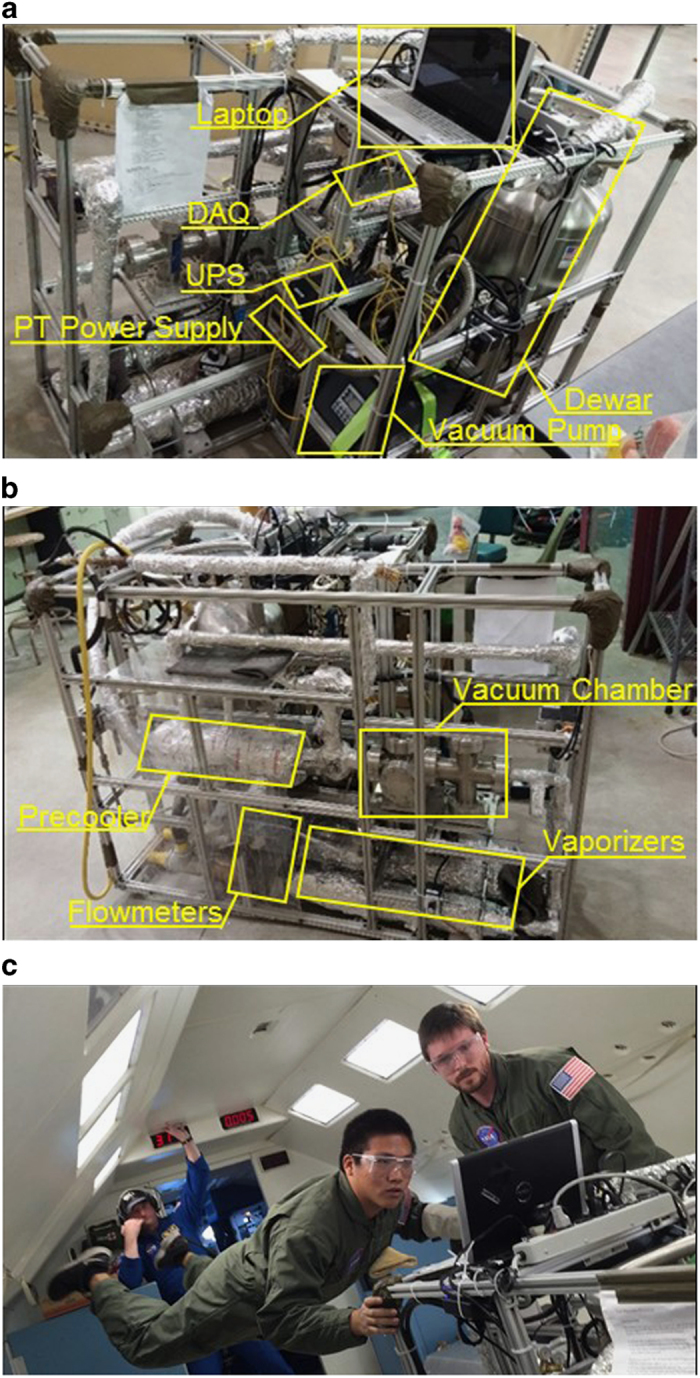
Flight experiment. (**a**) Front view of the rig showing the laptop used to collect data, the data acquisition unit (DAQ), the uninterrupted power supply (UPS) that was used to prevent loss of data in case of a power failure on the plane, the power supply for the pressure transducers, the liquid nitrogen dewar that held the test liquid, and the vacuum pump that vacuumed out the vacuum chamber; (**b**) side view of the rig showing the precooler, or subcooler, that cooled the plumbing upstream of the test section before each test, the vacuum chamber which housed the pipe test section equipped with thermocouples and pressure transducers, the two vaporizers which vaporized the two-phase mixture coming out of the test section, and the two flow meters that measured the flow rate history for each test; and (**c**) crew members at work during a parabolic maneuver.

This experiment was designed in much the same way as the experiment from Darr *et al*.^8^ with small modifications to make the rig flight-ready. Figure 4 shows pictures of the flight hardware. The apparatus weighed a total of 363 kg when the dewar was full of LN_2_. All the components of the apparatus fit within a 1.5 m long×0.76 m wide×0.91 m tall aluminum frame. Like the original apparatus, the new apparatus was designed so that (i) all pertinent thermodynamic and flow variables were measured or calculated with good accuracy (including local wall superheat, heat flux, mass flux, pressure, and equilibrium quality), (ii) the effect of axial distance on the chilldown process could be determined, and (iii) the test section was able to cool down completely so that a single-phase liquid flow regime existed at the end of each test.

### Experiment system design

**Figure 5:**
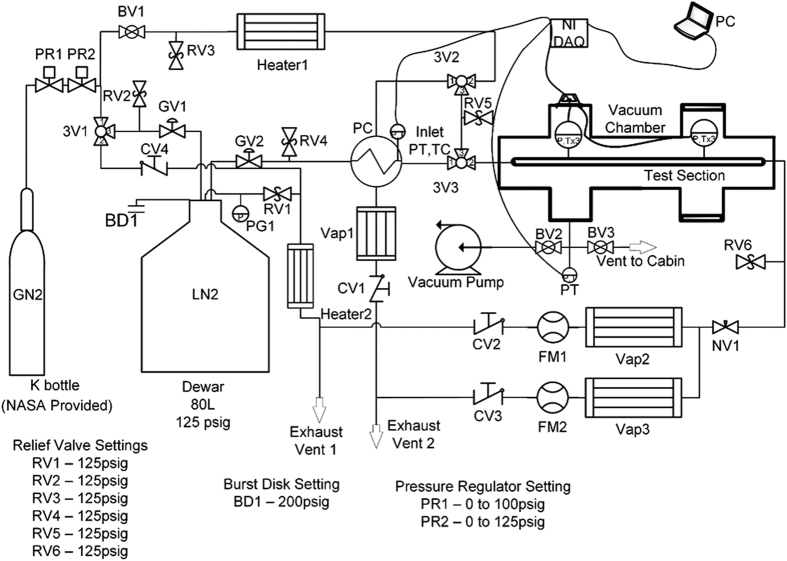
Fluid system schematic. The valves and important components of the fluid network. Relief valve settings, the burst disk setting, and pressure regulator settings are also included. BD, burst disk; BV, ball valve; CV, check valve; DAQ, data acquisition unit; FM, flow meter; GN_2_, gaseous nitrogen; GV, globe valve; LN_2_, liquid nitrogen; NASA, National Aeronautics and Space Administration; NV, needle valve; PG, pressure gauge; PR, pressure regulator; PT, pressure transducer; RV, relief valve; TC, thermocouple; Vap, vaporizer; 3V, three-way valve.

Reduced-gravity experiments were carried onboard a C9 aircraft for four flights and a total 160 parabolas, each parabola providing about 20–23 s of a ±0.02 g gravity level. The time between reduced-gravity periods was about 90 s. A fluid schematic of the experiment is shown in Figure 5. LN_2_ was supplied to the system from an 80 l double-walled dewar, with a relief valve set at 861 kPa. A gaseous nitrogen cylinder initially at 15 MPa pressurized the dewar to a set value for each test, which ranged between 90 and 830 kPa absolute pressure. Dewar pressure was managed by pressure regulator 1 (PR1), which fixed the dewar pressure to within 35 kPa of the set value during each test. Depressurization was carried out by opening globe valve 1 (GV1) and three-way ball valve 1 (3V1) to allow ullage gas to escape the dewar and vent overboard. Pressurization was carried out by closing ball valve (BV1) and opening BV2 to route flow from the first gas cylinder to the dewar. PR1 was used to regulate the dewar pressure. PR2 was a backup regulator to prevent large pressure spikes. In operation, PR2 was always left fully open to 862 kPa gauge pressure.

The dewar was used both for prechilling the plumbing upstream of the test section and for running the actual chilldown experiment. LN_2_ was delivered through a ½″ nominal inner diameter (ID) globe valve (GV2). GV2 was connected, through a 1.2 m long, 1.27 cm outside diameter (OD), 1.18 cm ID 304SS braided hose to the inner tube of a “subcooler”. Further details of the subcooler are given in Darr *et al.*^8^ The purpose of the subcooler was to insulate the tubing upstream of the test section so that the fluid entering the test section was vapor free, subcooled liquid.

During the prechilling process, the liquid exiting the inner tube of the subcooler was directed by two “T-type” 316SS ½″ ID three-way ball valves (3V3 and 3V2) to a fill port on top of the outer vessel of the subcooler. A 3 cm long, 1.270 cm OD, 1.168 cm ID 304SS tube connected 3V3 and the subcooler. The fill port and vent port of the subcooler were switched from the original experiment. A 2.5 cm ID port allowed evaporating liquid to escape the subcooler. The fluid was directed to an electrically heated “vaporizer” Vap1, which vaporized any entrained liquid and warmed the vapor above 273 K so that it could be vented overboard without freezing moisture in the air outside of the plane. A pressure transducer (PT) and thermocouple (TC), labeled “Inlet PT, TC” in the schematic, were connected at a distance of 7 cm from the downstream side of the inner tube of the subcooler to measure a fluid pressure and fluid temperature near the inlet of the test section, which determined the level of inlet subcooling for each test.

Once this temperature measurement reached a steady value, and the temperature was below the saturation temperature based on the measured pressure, a chilldown test could begin. To start a test, 3V3 was turned so that flow was directed from the dewar to the test section instead of the subcooler fill port. The valve was manually operated during flight. Flow entering 3V3 would turn 90° towards 3V2 during the subcooling operation, or it would travel straight through to flow into the test section during a test. This allowed the subcooler and the test section to be in line so that upstream of the test section was 57 cm of straight tube, much more than was necessary to create fully developed liquid flow. This was an improvement on the original experiment which used an “L”-port type valve that created a 90° bend just upstream of the test section inlet. The subcooler and test section were connected to the rig so that they were parallel to the plane floor, in a horizontal flow configuration. For most of the tests, the reduced-gravity quality was so good that there was little to no difference between the temperatures at the top and bottom of the test section. Two layers of 6.35 mm thick aerogel insulation were wrapped around GV2, the hose upstream of the subcooler, the subcooler itself, 3V3, and the 3 cm length of tube between 3V3 and the subcooler to minimize heat leak into the system upstream of the test section.

The test section was a 57.2 cm long, 1.270 cm OD, 1.168 cm ID 304SS tube. A length of 2.54 cm of the test section tube protruded out of each side of the vacuum chamber. Six TCs were soldered to the outside of the tube, with three placed an axial distance of 14.9 cm from the test section inlet (upstream TC station), and three placed 40.1 cm from the inlet (downstream TC station). Detailed drawings and dimensions are in Darr *et al.*^8^ For each station, the TCs were spaced out radially in 90° increments such that each station had a top, side, and bottom TC. However, due to an error when installing the system onto the frame of the flight rig, the TCs were skewed by an angle of approximately 10° from the designed location as shown in Figure 5. All three TCs at each station were recorded for the vertical tests and an average cross-section temperature at each time step was recorded. Two cryogenic rated PTs were connected to two short pieces of 304SS tube protruding perpendicularly from the test section, one near each TC station. These tubes were welded to the test section.

The 316SS vacuum chamber that housed the test section was the same as the original experiment. The purpose of the vacuum chamber was to reduce parasitic heat leak, which reduced the uncertainty in the calculation of wall-to-fluid heat flux. A diaphragm pump and molecular turbopump were used to bring the air pressure surrounding the test section to approximately 1 Pa. This reduced the parasitic heat leak due to conduction between the test section and the air inside the vacuum chamber to less than 10% of the lowest measured convective heat flux, which occurred during film boiling at the lowest flow rate that was tested. Parasitic heat leak was less than 1% of the measured value for most of the data points.

The needle valve downstream of the test section (NV1) was throttled at three different positions so that tests could be run at different flow rates for the same dewar pressure setting. The flow was routed from the needle valve by a stainless steel tube to two separate pipes (labeled Vap2 and Vap3) that were electrically heated to vaporize the flow. To enhance the heat transfer, eight 1.27 cm OD copper tubes were packed inside each pipe in an octaweb configuration. One electrical heating tape was wrapped around each pipe to heat it to 480 K before each test. A TC was placed on the outer surface of each heat tape to monitor the temperature in real time. The flow out of Vap2 and Vap3 entered two separate, identical gas flow meters (FM1 and FM2) each of which had a capacity of 4,000 standard liters per minute. The flow was then directed to the airplane vent ports.

The test was completed when the test section TC measurements read a steady-state temperature below the saturation temperature based on the test section PT readings. At this time, the liquid-delivery valve on the dewar, GV2, was closed to prevent liquid flow from the dewar. Before running the next test, the test section was quickly heated up by opening BV1 and turning 3V1 and 3V2 to allow warm gas flow from the gas cylinder to enter the test section. Once the dewar was pressurized and the test section was heated to room temperature, the next test was ready to begin with the subcooling process.

A data acquisition unit collected all sensor data and displayed it real time on a laptop computer connected to the rig. All TCs were T-type and were measured at a frequency of 15 Hz. The flow meter readings were measured at roughly 2 Hz, and the PT readings were measured at 80 Hz.

### Experimental procedure

The tests were operated as follows: At the start, the needle valve was set to the target position for the given test, Vap 1, Vap2, and Vap3 were heated up to 480 K, and the vacuum pump system was turned on. The total time from starting the pump to reaching 1 Pa inside the vacuum chamber took roughly 15 min. Concurrently, the inner tube inside the subcooler was chilled by pressurizing the dewar, opening GV2, and directing the flow through 3V3 and 3V2 to the fill port of the subcooler. The subcooler took approximately 10 min to completely chill and fill. Then, 3V2 was shut off from the flow from 3V3, and the dewar was pressurized by opening PR1 to the desired dewar gauge pressure. Pressurization was done as quickly as possible before the liquid inside the dewar could warm to the saturation temperature at the new dewar pressure, and also before the liquid inside the plumbing upstream of the test section could gain enough heat to start boiling. Shortly after, 3V3 was turned to start the flow into the test section. Once the TC readings dropped to or below the saturation temperature and maintained a steady temperature, GV2 was closed. This marked the end of the test. In preparation for the next test, Heater 1 was turned on and 3V2 and 3V3 were rearranged so that warm gas entered the test section to carry out the reheat process. After reheating finished, NV1 was set at the new position. Vap2 and Vap3 were allowed to heat up to above 480 K, and the subcooling process was repeated to account for the LN_2_ that had been lost to evaporation.

About four to five tests per flight were achievable with this procedure. As each flight consisted of 40 parabolas over about 2 h, many of the parabolas were spent in preparation for the next test. Owing to the unexpected behavior of the system in the changing gravity conditions during test preparation, only two or three tests per flight were obtained.

## Figures and Tables

**Table 1:** Test data summary

*Flow configuration*	*Case number*	*Inlet liquid Re*	*Pressure at PT-1 (kPa)*	*Inlet subcooling (K)*	*Mass flux (kg/m*^*2*^•*s)*
Reduced gravity	1	2,838	169.93	0	31.31
	2	6,145	242.04	0	67.77
	3	8,801	100.31	0	116.17
	4	12,133	135.28	0	143.76
	5	13,318	126.00	0	166.22
	6	13,881	288.24	0	130.42
	7	28,704	317.63	0	262.26
	8	35,291	365.48	0	304.38
	9	46,245	441.74	0	375.26
	10	74,177	711.85	0	503.06
					
+90° Vertical upward	1	2,888	259.65	0	28.45
	2	4,841	202.40	0	50.80
	3	7,421	188.57	0	84.84
	4	11,097	163.15	0	140.91
	5	—	—	—	—
	6	11,349	275.61	0	126.05
	7	33,985	294.97	0	316.65
	8	35,254	327.95	0	334.47
	9	45,059	375.23	1.95	400.93
	10	60,327	446.72	1.16	449.85
					
0° Horizontal	1	2,059	149.13	0	24.93
	2	5,483	251.10	0	53.96
	3	7,525	210.95	0	81.45
	4	—	—	—	—
	5	—	—	—	—
	6	12,366	267.17	0	121.06
	7	31,000	312.30	0.79	294.82
	8	38,103	344.46	1.60	344.04
	9	47,623	403.69	0.76	403.10
	10	84,155	635.22	7.36	612.14
					
−90° Vertical downward	1	2,116	194.77	0	23.21
	2	—	—	—	—
	3	5,678	135.22	0	68.77
	4	—	—	—	—
	5	—	—	—	—
	6	14,172	302.04	0	127.56
	7	34,377	295.54	0	315.87
	8	36,504	333.66	0	325.69
	9	52,361	365.21	2.33	426.40
	10	73,953	714.00	4.87	477.64

Flow conditions for each reduced gravity test and counter ground test. All the values are time-averaged over the entire reduced-gravity period.

**Table 2:** Film boiling correlation coefficients

*Flow configuration*	*c*_*1*_	*c*_*2*_	*c*_*3*_	*c*_*4*_	*c*_*5*_	*c*_*6*_	*c*_*7*_
Reduced gravity	0.150	−0.76	0.52	5E−6	0.10	2	0
+90° Vertical upward	0.112	−0.80	0.57	7E−5	0.45	2	0.06
−90° Vertical downward	0.112	−0.80	0.57	7E−5	0.45	2	−0.25

Coefficients for equations (2, 3, 4).

## Notes

Supplementary Excel File
